# Neurotoxicity of novel cancer immunotherapies

**DOI:** 10.1007/s00415-019-09444-4

**Published:** 2019-06-28

**Authors:** M. D. Willis, N. P. Robertson

**Affiliations:** 1 Department of Neurology, University Hospital Wales, Cardiff, CF14 4XN UK; 2 Institute of Psychological Medicine and Clinical Neuroscience, Cardiff University, University Hospital of Wales, Heath Park, Cardiff, C14 4XN UK

## Introduction

Novel immunotherapies are emerging as an exciting treatment for a variety of haematological and solid malignancies. Chimeric antigen receptor (CAR) T cells and immune checkpoint inhibitors (ICPI) are the two classes of treatment currently available. Despite very promising oncological outcomes for these treatments, neurotoxicity has emerged as a serious adverse event. With a growing number of oncological indications, clinicians now need to be aware of the neurological side effects of these therapies.

CAR T cell therapy involves the genetic engineering, via viral transduction, of the patients own T cell population, to incorporate antigen-recognition moieties as well as T cell activation costimulatory domains. Following apheresis, viral transduction and expansion ex vivo, the CAR T cell population is re-infused into the patient, which then directs to the desired target antigen on the cancer cell surface. The CAR T cells are then activated without the need for MHC–epitope presentation or antigen presenting cell co-stimulation, with the aim of elimination of the cancer. Currently, there are two licenced CAR T cell therapies in the US and Europe for relapsed or refractory acute lymphoblastic leukaemia and B-cell lymphoma. However, it is likely that the indication for these therapies will soon expand to include a wide range of other malignancies. Following treatment with CAR T cells, neurotoxicity affects up to 60% of treated patients and commonly presents with encephalopathy, although tremor, seizures, and cerebral oedema may also feature. The pathogenesis of the neurotoxicity remains unclear but enrichment of pro-inflammatory cytokines in the central nervous system (CNS), endothelial activation, and macrophage activation syndrome have all been proposed. Cytokine release syndrome (CRS) may also be seen as CAR T cells encounter their target cells. This clinical syndrome may resemble sepsis and can lead to multiple organ failure and death in severe cases.

Immune checkpoints are utilised by the body to inhibit T-cell activation, maintain self-tolerance and prevent autoimmunity. However, cancer cells can commandeer this pathway to evade immune detection. ICPIs act to remove this immune brake and as a result increase the T cell response against the cancer. Monoclonal antibodies against cytotoxic lymphocyte-associated protein 4 (CTLA-4), programmed cell death 1 (PD-1) and PD ligand 1 (PD-L1) have been the first therapies approved for a variety of malignancies. In contrast to treatment with CAR T cells, neurotoxicity related to ICPIs is rare, but has wide and varied neurological presentations involving both the peripheral and central nervous system. This toxicity is thought to occur as a result of T cell activation leading to autoimmune pathology.

Because of the limited clinical experience of using these therapies, optimal definitions, grading systems and management strategies are still to be fully optimised. This month's journal club outlines three papers that provide further descriptions of the neurological side effects of these promising therapies.

## ASTCT consensus grading for cytokine release syndrome and neurologic toxicity associated with immune effector cells

The assessment and grading of CRS and neurotoxicity associated with novel immunotherapy vary across clinical institutions and trials. Historically, different terms have been used for similar symptoms with grading systems based on the ability to perform functional activities, which may not be practical for bedside application. To compare safety profiles and develop optimal management, it is therefore of importance to develop a standardised grading criteria. In this regard, this paper presents expert consensus guidelines supported by the American Society for Transplantation and Cellular Therapy (ASTCT).

First, the group defines CRS as “a supraphysiologic response following immune therapy that results in the activation or engagement of endogenous or infused T cells and/or other immune effector cells. Symptoms can be progressive, must include fever at the onset, and may include hypotension, capillary leak (hypoxia) and end organ dysfunction”. Importantly, they note that other aetiologies for the patient’s symptoms must be excluded and that CRS rarely presents beyond 14 days after initiation of therapy. The group then define 5 grades of CRS, all of which feature fever (≥ 38.0 °C) and are dependent on the presence or absence of constitutional symptoms (myalgia, arthralgia, malaise), hypotension (including vasopressor requirement) and hypoxia (including oxygen requirements). Grade 5 is defined as death due to CRS. Although biomarkers were excluded from the definition and grading of CRS, the authors encourage their use (cytokines, CRP, ferritin) to inform future studies.

Neurotoxicity (termed ICANS; immune effector cell-associated neurotoxicity syndrome) is then defined as “a disorder characterised by a pathologic process involving the central nervous system following any immune therapy that results in the activation or engagement of endogenous or infused T cells and/or other immune effector cells. Symptoms or signs can be progressive and may include aphasia, altered level of consciousness, impairment of cognitive skills, motor weakness, seizures and cerebral oedema”. In the authors' grading of ICANS, an immune effector cell-associated encephalopathy (ICE) score is incorporated, which then with the presence or absence of other neurological symptoms (level of consciousness, seizures, motor symptoms and elevated ICP/cerebral oedema) gives an overall ICANS grade.

Comment: this paper robustly sets out the definitions and grading criteria for CRS and neurotoxicity associated with novel immunotherapies used to treat malignancies. Use of this grading system will allow comparison between centres and trials and further the knowledge around these treatments. They emphasise that despite the agreed guidelines, further data should be collected due to the limited number of treated patients across clinical trials. This will then inform further clinical guidelines.

*Lee et al. Biol Blood Marrow Transplant. 2019;25(4):625–638.*

## Clinical presentation, management, and biomarkers of neurotoxicity after adoptive immunotherapy with CAR T cells

This case series details 25 patients who developed CAR T-cell-mediated CRS and neurotoxicity following treatment for a variety of haematological malignancies, and hepatocellular carcinoma. The overall disease response rate after CAR T cell infusion was 84% and median overall survival 54.7 weeks. 12/25 developed low-grade (LGNT) and 13/25 high-grade neurotoxicity (HGNT) with the median time to first symptoms of 5 ± 0.9 days. By the date of database cutoff, 8/13 patients with HGNT had died with no deaths in the low-grade group.

In those with LGNT, CRS was present in 92% and always preceded the onset of neurological symptoms, which consisted of encephalopathy (92%), aphasia (50%) and tremor (33%). Median duration of symptoms was 5 ± 1.8 days. All 13 patients with HGNT experienced severe encephalopathy, with involuntary movements suggestive of seizure activity or nonepileptic myoclonus seen in a majority (77%). Symptoms often remained present > 30 days after CAR T-cell infusion or were ended by patient death. All HGNT patients developed CRS, although neurotoxicity preceded its onset in two patients. Both CRP and ferritin levels rose following CAR T-cell infusion. Absolute peak concentrations for ferritin were significantly more elevated in patients with HGNT compared to those with LGNT and normalised when symptoms resolved. Platelet counts were significantly lower at the time of CAR T-cell infusion in patients who later developed HGNT and may be a marker of blood–brain barrier disruption.

Of 10 patients who were imaged with brain MRI (LGNT *n* = 2, HGNT = 8) only 2 patients, both with high-grade symptoms, had new hyperintense lesions situated in the periventricular white matter (*n* = 2), splenium (*n* = 2) and pons (*n* = 1). EEG was performed on 18 patients (6 low- and 12 high-grade) with diffuse or frontal background slowing present in all. Periodic or rhythmic patterns within the ictal–interictal continuum were seen in 50% and 92% of low- and high-grade patients, respectively. Management for mild neurotoxicity included supportive therapy, and tocilizumab (IL-6R monoclonal antibody) for patients with CRS. Most patients received levetiracetam either as prophylaxis or at first neurologic symptoms with additional antiepileptics used for abnormal EEG patterns and status epilepticus. Dexamethasone was commonly used to treat neurotoxicity.

Comment: this paper informs clinicians of the presentation and management of patients with neurotoxicity following CAR T cell therapy. It is important to note that HGNT, age > 65 years and a prolonged course of steroids (> 10 days) were significant negative markers for overall survival and ferritin may be helpful as a biomarker to follow disease course. In addition, steroid use ≥ 7 days did not alter outcome when compared with < 7 days of treatment. Further studies will be required to further clarify the clinical spectrum of neurotoxicity and decide on optimal management, particularly with regard to different products as and when they reach the bedside.

*Karschnia P et al. Blood. 2019;133:2212–2221.*

## Neurologic complications of immune checkpoint inhibitors

Neurotoxicity following immune checkpoint inhibitors (ICPI) is less common than with CAR T cell therapy, with a reported incidence of 3.8%, 6.1%, and 12%, respectively, for anti-CTLA4 antibodies, anti-PD1 antibodies and when used in combination. Most symptoms are considered low-grade including headache, dysgeusia, dizziness and paraesthesia. Although high-grade adverse events are only observed in < 1% of cases they include a broad range of diseases affecting both the central and peripheral nervous systems. There are limited data regarding ICPI-related neurotoxicity, which this paper tries to address by presenting a retrospective review of nine patients who experienced neurological adverse events following ICPI treatment.

Patients included in this study had a variety of primary malignancies: renal cell carcinoma, melanoma, Hodgkin lymphoma and glioblastoma. Four patients were treated with a combination of checkpoint inhibitors (anti-PD1 and anti-CTLA4). Median time to onset of neurotoxicity was 8 weeks (5 days–19 weeks) with a variety of diagnoses observed including meningoencephalitis (*n* = 2), polyradiculitis (*n* = 2), limbic encephalitis (*n* = 1), myositis (*n* = 1), ocular myasthenic syndrome (*n* = 1), reactivated myasthenia (*n* = 1) and cranial polyneuropathy (*n* = 1). Detailed clinical accounts are only given for three patients in the paper.

Following the onset of neurotoxicity, steroids were commenced in all patients and ICPI treatment stopped. 6/9 patients made a full recovery, one patient a partial recovery and two patients died. The patient who experienced myositis had a partial response to steroids but then died of cardiac complications. The patient with a myasthenic syndrome did not receive any benefit with steroids or plasmapheresis and died of septic shock. Interestingly, this patient had a remote history of ocular myasthenia that had not required treatment for several years prior to ICPI therapy. In two patients, ICPI treatment was recommenced following resolution of neurological symptoms, with no further adverse events.

Comment: this paper provides an example of the wide variety of neurological diseases that can potentially develop post ICPI therapy. Because of the rarity of these adverse events, the authors recommend making a diagnosis of ICPI-related neurotoxicity after excluding other potential causes including infectious, metabolic, paraneoplastic and neoplastic aetiologies. Although it is difficult to confirm a definitive diagnosis, they also emphasise the importance of recognising neurotoxicity as quickly as possible so that appropriate treatment can be initiated. Of note, once steroids have been commenced it is recommended that they be slowly tapered over 2–3 months due to the long half-life of ICPI therapy. Other immunomodulatory and immunosuppressive treatments may be required in steroid-resistant cases.

*Fellner et al. J Neurooncol. 2018;137(3):601–609.*

